# Supplementary figures and images for: Significance of skull osteoporosis to the development of peritumoral brain edema after LINAC-based radiation treatment in patients with intracranial meningioma
Source: PLoS One. 2020 Feb 18;15(2):e0226312. doi: 10.1371/journal.pone.0226312 (PMC7028281; doi:10.1371/journal.pone.0226312)

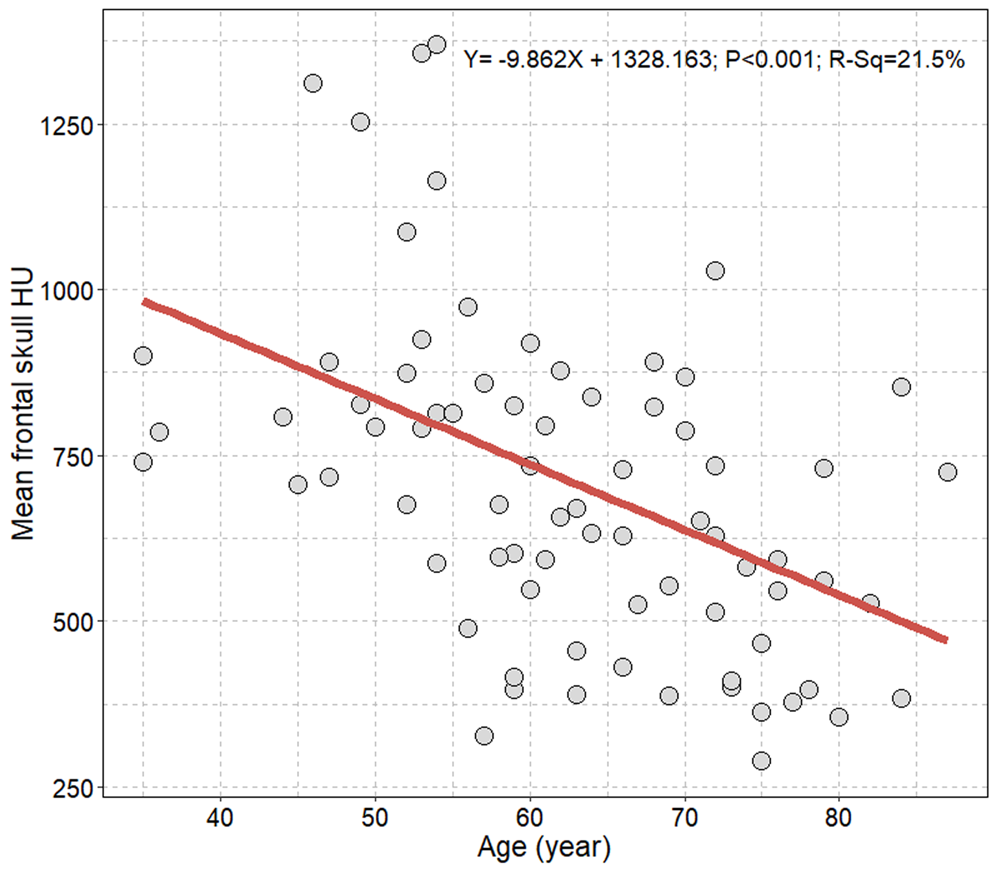

Supplement: S1 Fig — HU = Hounsfield unit. (TIF) [file pone.0226312.s001.tif]
