# Supplementary material for: Significance of skull osteoporosis to the development of peritumoral brain edema after LINAC-based radiation treatment in patients with intracranial meningioma
Source: PLoS One. 2020 Feb 18;15(2):e0226312. doi: 10.1371/journal.pone.0226312 (PMC7028281; doi:10.1371/journal.pone.0226312)
Supplement: S1 Table — (DOCX) [file pone.0226312.s002.docx]

|  | Univariate analysis | | Multivariate analysis | |
| --- | --- | --- | --- | --- |
| Variable | HR (95% CI) | P value | HR (95% CI) | P value |
| Sex |  |  |  |  |
| Male | Reference |  | Reference |  |
| Female | 1.27 (0.28–5.80) | 0.759 | 0.96 (0.18–5.30) | 0.967 |
| Age (per 1-year increase) | 1.09 (1.03–1.16) | 0.002 | 1.05 (0.97–1.13) | 0.229 |
| BMI (per 1 BMI increase) | 0.95 (0.80–1.14) | 0.610 | 0.98 (0.77–1.25) | 0.889 |
| Mean frontal skull HU |  |  |  |  |
| ≤630.6 | 9.83 (2.13–45.23) | 0.003 | 7.04 (1.16–42.85) | 0.034 |
| >630.6 | Reference |  | Reference |  |
| GTV |  |  |  |  |
| ≤7.2 cc | Reference |  | Reference |  |
| >7.2 cc | 4.17 (1.27–13.74) | 0.019 | 7.20 (1.37–37.81) | 0.020 |
| Location |  |  |  |  |
| Convexity | 2.41 (0.74–7.88) | 0.145 | 1.89 (0.50–7.11) | 0.348 |
| Other regions | Reference |  | Reference |  |
| BED (α/β=3)  (per 1-Gy increase) | 1.01 (0.97–1.04) | 0.725 | 1.00 (0.95–1.05) | 0.888 |
| Fractionation  (per 1-fraction increase) | 0.92 (0.82–1.04) | 0.184 | 0.72 (0.43–1.22) | 0.221 |

HR, hazard ratio; CI, confidence interval; BMI, body mass index; HU, Hounsfield unit; GTV, gross tumor volume; BED, biologically equivalent dose
