# Supplementary material for: Significance of skull osteoporosis to the development of peritumoral brain edema after LINAC-based radiation treatment in patients with intracranial meningioma
Source: PLoS One. 2020 Feb 18;15(2):e0226312. doi: 10.1371/journal.pone.0226312 (PMC7028281; doi:10.1371/journal.pone.0226312)
Supplement: S2 Table — (DOCX) [file pone.0226312.s003.docx]

|  | Univariate analysis | | Multivariate analysis | |
| --- | --- | --- | --- | --- |
| Variable | HR (95% CI) | P value | HR (95% CI) | P value |
| Sex |  |  |  |  |
| Male | Reference |  | Reference |  |
| Female | 1.27 (0.28–5.80) | 0.759 | 1.06 (0.19–6.02) | 0.950 |
| Age (per 1-year increase) | 1.09 (1.03–1.16) | 0.002 | 1.06 (0.98–1.15) | 0.130 |
| BMI (per 1 BMI increase) | 0.95 (0.80–1.14) | 0.610 | 1.03 (0.80–1.33) | 0.814 |
| Mean frontal skull HU |  |  |  |  |
| ≤630.6 | 9.83 (2.13–45.23) | 0.003 | 9.41 (1.45–61.16) | 0.019 |
| >630.6 | Reference |  | Reference |  |
| GTV |  |  |  |  |
| ≤7.2 cc | Reference |  | Reference |  |
| >7.2 cc | 4.17 (1.27–13.74) | 0.019 | 6.02 (1.06–34.06) | 0.042 |
| Location |  |  |  |  |
| Convexity | 2.41 (0.74–7.88) | 0.145 | 1.65 (0.43–6.35) | 0.465 |
| Other regions | Reference |  | Reference |  |
| BED (α/β=3)  (per 1-Gy increase) | 1.01 (0.97–1.04) | 0.725 | 1.01 (0.96–1.06) | 0.646 |
| Fractionation  (per 1-fraction increase) | 0.92 (0.82–1.04) | 0.184 | 0.77 (0.45–1.31) | 0.326 |
| Past medical history |  |  |  |  |
| Hypertension | 1.12 (0.37–3.39) | 0.837 | 0.44 (0.10–1.88) | 0.266 |
| Diabetes | 1.12 (0.30–4.12) | 0.870 | 0.57 (0.12–2.73) | 0.483 |

HR, hazard ratio; CI, confidence interval; BMI, body mass index; HU, Hounsfield unit; GTV, gross tumor volume; BED, biologically equivalent dose
